# Supplementary material for: Impact of Friendship Bench problem-solving therapy on adherence to ART in young people living with HIV in Zimbabwe: A qualitative study
Source: PLoS One. 2021 Apr 22;16(4):e0250074. doi: 10.1371/journal.pone.0250074 (PMC8061927; doi:10.1371/journal.pone.0250074)
Supplement: S1 File — (DOCX) [file pone.0250074.s001.docx]

**S1 File. Interview topic guide Shona**

Ongororo ino irikuda kuona kubatsira kuri kuita hurukuro yepaFriendship Bench pamaonero nematorero amurikuita HIV.

Ndirikuda kunzwisisa kuti makagamuchira sei rubatsiro rwepaFriendship Bench uye nekuona matambudziko amakasangana nawo mumashure neamuchiri kusangana nawo pakutora mushonga weART sekurairidzwa kwamakaita.

Ndisati ndakubvunzai mibvunzo ndichatanga ndakuverengerai gwaro retenderano. Inzwai makasununguka kubvunza mubvunzo yamunenge munayo maererano negwaro iri. Makasununguka kusapindura chero upi mubvunzo wamusina kusununguka nawo. Hamuzomanikidzwa kupindura mibvunzo yese.

**Mibvunzo:**

1. *Chakaita kuti vaongororwi vauye kuFriendship Bench nezvavaitarisira*

- Chii chakaita kuti muuye ku Friendship Bench?
- Chii chamaitarisira kubva kubhenji rehushamwari?

1. *Ndinoda kuziva nezveupenyu hwenyu musati mauya kubhenji rehushamwari uye ruzivo rwenyu nezvekufungisisa*

- Ndiudzei nezvehupenyu hwenyu musati mauya kubhenji rehushamwari
  - Mungati maiswera muchiitei?
  - Ndevapi vanhu vainge vakakukosherai muhupenyu?
  - Zvii zvamaikoshesa muhupenyu ?
- Mainzwa sei nguva iyoyo
- Mungati maive nekufungisisa here? Nditsanangurirei

1. *Ndinoda kunzwisisa zvakaitika pamakaziva HIV status yenyu*

- Makanzwa sei pamakatanga kuzviziva kuti mune HIV?
  - Pave nenguva yakareba sei mazviziva?
  - Kurarama neHIV kwashandura mararamiro ehupenyu hwenyu here?
- Pane vemhuri kana shamwari vamakamboudza HIV status yenyu here? (Bvunza nezve mudiwa kana aine waari kufambidzana naye)
  - Makavaudzirei kana kuti makaregererei?
  - Kune vamakaudza, vakazvitora sei?
  - Makanzwa sei nezvazvo?
- Pane rubatsiro rwamakambowana maringe nezve vanorarama neHIV?

1. *Zvamakasangana nazvo pakutora mushonga weART musati mauya pabhenji rehushamwari*
2. *Kana vakatanga mushonga mushure mekuziva HIV status*

- Pamakatanga kutora mushonga mainzwa sei?
  - Chii chakaita kuti mutange kutora mushonga weART?
  - Chii chamunoziva pamusoro pemushonga weART?
  - Chii chamunoziva pamusoro peCD4 count neHIV viral load
- Ndinokumbirawo kuziva nezvekutora kwenyu mushonga weART.
  - Munonzwa sei?
  - Hupenyu hwenyu wakashanduka here nekutora mushonga?
    - (Kana ati hongu) Sei?
  - Munotora mushonga kangani?
    - *(Kana achiratidza kusatora mushonga zvakanaka)* Chii chinokutadzisai kutora mushonga sezvakatarwa?
    - Chii chinokubatsirai kutora mushonga?
    - Chii chingaite kuti urambe uchitora mushonga sezvakatarwa?

1. *Kana vanga vari paART vasati vaziva HIV status yavo (Kutapurirwa kwehutachiwana kubva kunamai kuenda kumwana)*

- Ndirikuedza kunzwisisa matorero amunoita mushonga weART
  - Chii chamunoziva pamusoro pemushonga iyi?
  - Chii chamunoziva pamusoro peCD4 count neviral load?
- Kana anga achitora ART asati aziva mamiriro ake maringe neHIV
  - Unonzwa sei nematorero enyu mushonga?
  - Unoutora kangani?
    - *(Kana mhinduro ichitaridza kusatora mushonga weART zvakanaka)* Chii chinokutadzisai kutora mushonga weART sezvakatarwa?
    - Chii chinokubatsira pakutora mushonga weART?
    - Chii chingaite kuti urambe uchitora mushonga sezvakatarwa?
- Mushure mekuziva mamiriro ako maringe neHIV, pane chakashanduka pakutora mushonga?
  - - *(Kana ati hongu)* Sei?

1. *Ndinoda kunzwisisa zvamakasangana nazvo uye nemaonero enyu rubatsiro rwepaFriendship Bench*

- Ndiudzeiwo nezvamakasamgana nazvo pabhenji rehushamwari
  - Chii chinoitika muhurukuro?
  - Mainzwanana here na ambuya?
  - Makataura nezvei?
    - Ndiudzeiwo hurukuro imwe yakanga yakakukosherai
- Ndiri kuedza kunzwisisa rubatsiro runounzwa nebhenji rehushamwari
  - Muri kunzwa sei mazuva ano?
  - Mungadaro muchine kufungisisa?
    - *(Kana mhinduro yakasiyana nemu section 1)* Munofungira kuti chii chakonzera shanduko?
  - Pane zvashanduka here muhupenyu hwenyu?
    - *(Kana ati Hongu)* Chii chakashandurwa nebhenji rehushamwari?
- Hurukuro yepaFriendship Bench ingasimudzirwa sei?

1. *Shanduko yematorero emushonga mushure mekupihwa rubatsiro rwepaBhenji rehushamwari?*

- Makambokurukura here pamusoro pezvamunosangana nazvo maererano neHIV pabhenji rehushamwari?
- *(Kana ati hongu)* Makataura nezvei?
- Makataura here nezvekutora mushonga weART?
- Wainzwa sei pamaita hurukuro?
- Pane zvitsva zvamakadzidza?
- Pane nzira dzamakawana here dzingakubatsirei pakutora mushonga sezvakatarwa?
- Makawana zvekukusimudzirai sevanhu vanorarama nehutachiwana hwe HIV?
- Makaona shanduko pakutora kwenyu mushonga mushure mekunge mapedza hurukuro dzepabhenji?
- *(Kana ati hongu)* Tsanangura shanduko dzacho
