# Supplementary material for: Impact of Friendship Bench problem-solving therapy on adherence to ART in young people living with HIV in Zimbabwe: A qualitative study
Source: PLoS One. 2021 Apr 22;16(4):e0250074. doi: 10.1371/journal.pone.0250074 (PMC8061927; doi:10.1371/journal.pone.0250074)
Supplement: S2 File — (DOCX) [file pone.0250074.s002.docx]

**S2 File. Interview topic guide English**

**Introduction:**

This study is exploring the impact of the Friendship Bench counselling service on people’s mood and experience of HIV.

I’m here to understand how you felt throughout the counselling sessions received at the Friendship Bench, and to explore any difficulties you may have experienced in the past or are still experiencing in terms of taking your ART as prescribed.

Before starting the interview, I will take you through a consent form, please take as much time as needed and feel free to ask any questions on this document.

During the interview, if at any moment you’re not comfortable with a question, simply let me know. You have no obligation to answer any questions if you don’t want to, and we can also stop the interview at any moment if you don’t want to complete it anymore.

**Guiding questions:**

1. *What triggered them to visit the Friendship Bench and their initial expectations*

- What motivated you to attend the Friendship Bench?
- What were your expectations from the Friendship Bench?

1. *As a follow up from the previous question, have a sense about their life before coming to the Friendship Bench, and their feeling of depression*

- Tell me about you and your life before coming to the Friendship Bench.
  - Describe a typical day
  - Who are the important people in your life?
  - What were your major concerns?
- How was your mood back then?
- Do you feel you were depressed?
  - How did depression feel like, could you describe it?

1. *Participants’ experience about finding out HIV status*

- How did you feel when you first found out you were HIV positive?
  - How old were you?
  - Has HIV changed your life?
- Have you disclosed your HIV status to some family members and/or friends? *(ask specifically about their partner if they are in a relationship)*
  - Why? Or why not?
  - *(If yes)* How did they react?
  - How did it make you feel?
- Did you find any sources of support for people living with HIV?

1. *Experience with ART and any difficulties they were experiencing with adherence before the Friendship Bench*
2. *If they started ART after finding out about their HIV status*

- I’m trying to understand how you felt when you first started taking medicine to treat HIV/AIDS (ART).
  - How did you decide to start ART?
  - What do you know about ART?
  - What do you know about CD4 count and HIV viral load?
- Please tell me about your overall experience of taking ART.
  - How do you feel about it?
  - Has your daily life changed to take ART?
    - *(If yes)* How?
  - How often do you take it?
    - *(If reply indicates nonadherence)* What makes it difficult to take your ART as prescribed?
    - What helps you take your ART?
    - What do you feel could help you further to take your ART as prescribed?

1. *If they were on ART before knowing their HIV status (mother-to-child transmission of HIV)*

- I’m trying to understand your overall experience of taking ART.
  - What do you know about ART?
  - What do you know about CD4 count and HIV viral load?
- You have been taking ART most of your life, before finding out about your HIV status.
  - How do you feel about it?
  - How often do you take it?
    - *(If reply indicates nonadherence)* What makes it difficult to take your ART as prescribed?
    - What helps you take your ART?
    - What do you feel could help you further to take your ART as prescribed?
  - After finding out about your HIV status, has anything changed in your experience taking ART?
    - *(If yes)* How?

1. *Overall experience through the Friendship-Bench counselling sessions and their perception of any changes in their mental well-being and quality of life*

- Please tell me about your experience at the Friendship Bench
  - What happens during a counselling session?
  - How was your interaction with the counsellor?
    - Do you always meet with the same person?
  - What do you talk about?
    - Tell me about one discussion that was most important to you
- I’m trying to understand the impact of the Friendship Bench counselling. How do you feel these days?
  - How is your mood?
  - Do you feel depressed?
    - *(If reply is different than in section 1)* What do you think has impacted on the change?
  - Has anything changed in your life?
    - *(If yes)* What are you doing differently as a result of the Friendship Bench?
- How could the counselling provided at the Friendship Bench be improved?

1. *Perception of any changes in adherence during/following the Friendship Bench therapy*

- Have you talked about your experience with HIV at the Friendship Bench?
  - *(If yes)* What did you talk about?
- Have you spoken specifically about taking ART?
  - How did you feel during those discussions?
  - Have you learned anything new?
  - Have you identified some ways to help you take your ART as prescribed?
- Have you identified additional sources of support for people living with HIV?
- Have you noticed any changes with taking ART after finishing your counselling at the Friendship Bench?
  - (If yes) Please describe those changes
